# Farming practices change food web structures in cereal aphid–parasitoid–hyperparasitoid communities

**DOI:** 10.1007/s00442-012-2387-8

**Published:** 2012-06-27

**Authors:** Katharina Lohaus, Stefan Vidal, Carsten Thies

**Affiliations:** 1 Department of Crop Sciences, Entomology, Georg-August-University Göttingen, Grisebachstraße 6, 37077 Göttingen, Germany; 2 Department of Crop Sciences, Agroecology, Georg-August-University Göttingen, Griesebachstraße 6, 37077 Göttingen, Germany

**Keywords:** Agricultural intensification, Organic farming practices, Biodiversity, Interaction structures, *Sitobion avenae*

## Abstract

Agricultural intensification has been shown to result in a decline in biodiversity across many taxa, but the changes in community structure and species interactions remain little understood. We have analysed and compared the structure of feeding interactions for cereal aphids and their primary and secondary parasitoids in organically and conventionally managed winter wheat fields using quantitative food web metrics (interaction evenness, generality, vulnerability, link density). Despite little variation in the richness of each trophic group, food web structures between the two farming systems differed remarkably. In contrast to common expectations, aphids and primary parasitoids were characterized by (1) a higher evenness of interaction frequencies (interaction evenness) in conventional fields, which cascaded to interactions at the next trophic level, with (2) a higher interaction evenness, (3) a higher ratio of primary parasitoid taxa per secondary parasitoid (generality) and (4) a higher link density. Aphid communities in the organically managed fields almost exclusively consisted of a single ear-colonizing species, *Sitobion avenae*, while highly fertilized conventional fields were mainly infested by leaf-colonizing aphids that benefit from the nutritional status of winter wheat. In conclusion, agricultural intensification appears to foster the complexity of aphid–parasitoid food webs, thereby not supporting the general expectation on the importance of organic farming practices for species richness and food web complexity.

**Electronic supplementary material:**

The online version of this article (doi:10.1007/s00442-012-2387-8) contains supplementary material, which is available to authorized users.

## Introduction

The structure of ecological communities is characterized by trophic interactions, particularly by the existence of feeding links and the strength of interactions among species. Such networks of feeding interactions may be of elemental importance for the functioning of ecological processes, such as biological control (van Veen et al. 2006; Tylianakis et al. 2010). In agricultural landscapes, increased applications of fertilizers and pesticides have led to a degradation of habitats and losses in the biodiversity of several taxonomic groups. Organic farming, as an alternative to conventional farming, has been suggested to counteract such changes in community structure and function (reviewed by Bengtsson et al. 2005; Hole et al. 2005; Kasperczyk and Knickel 2006). Additionally, the insurance hypothesis predicts that a high diversity of natural enemies ensures the functioning of biological control because the larger number of species provide a greater guarantee that some species will maintain functioning if others fail in situations of environmental fluctuations (Yachi and Loreau 1999). The mechanisms of such effects, however, may not only be determined by diversity loss per se, but also by the identity of species that are becoming extinct (Cardinale et al. 2006).

The major cereal aphids in Europe, *Sitobion avenae* (Fabricius), *Metopolophium dirhodum* (Walker) and *Rhopalosiphum padi* (Linnaeus), are attacked by hymenopterous (primary) parasitoids belonging to the families of Braconidae (Ichneumonoidea) and Aphelinidae (Chalcidoidea) (Stary 1970), which in turn are parasitized by two guilds of (secondary) hyperparasitoids. These hyperparasitoids differ in their modality of host use. True hyperparasitoids of the subfamily Alloxystinae (Cynipoidea) attack primary parasitoids in the living aphid, delaying their development until the primary parasitoid has caused mummification (koinobiont strategy). Conversely, mummy parasitoids of the families Megaspilidae and Ceraphronidae (Ceraphronoidea), Pteromalidae and Encyrtidae (Chalcidoidea) attack the already mummified aphid, regardless of whether it contains a primary or secondary parasitoid, and develop without delay (idiobiont strategy). In our study, the koinobionts are represented by the genera *Alloxysta* and *Phaenoglyphis* and the idiobionts by the genera *Dendrocerus*, *Aphanogmus*, *Asaphes*, *Pachyneuron*, *Coruna* and *Aphidencyrtus*.

In our study, we constructed quantitative food webs of three taxa of cereal aphid pests, their primary parasitoids and secondary parasitoids in organically and conventionally managed winter wheat fields, respectively, in Northern Germany. The feeding interactions between cereal aphids and their parasitoids were investigated in a temporal series ranging from wheat flowering (the main period of aphid colonization in the fields) to wheat peak ripening (the period of aphid population breakdown owing to decreased resource quality). By resolving food webs to the genus level of insects, we were able to demonstrate interaction webs up to the fourth trophic level because we analysed the interactions between aphids (second trophic level) and primary parasitoids (third trophic level) as well as those between primary parasitoids and secondary parasitoids (fourth trophic level). Research on quantitative food webs is just beginning, but findings have revealed that community and interaction structures might not be detected in studies that focus simply on species richness and abundance (Albrecht et al. 2007; Tylianakis et al. 2007; Bukovinszky et al. 2008). Food web interactions have been studied for multiple species assemblages on organic and conventional farms (Macfadyen et al. 2009a) and for aphid–parasitoid systems in the landscape context (Gagic et al. 2011), but they have not explicitly explored to date in studies focussing on aphid–parasitoid interactions in organically versus conventionally managed fields. A main difference in farming practices between the two approaches is the relatively lower nitrogen input in organically managed farms (Maeder et al. 2002). The positive effects of increasing nitrogen inputs (host plant quality) on the abundance of cereal aphids (Homoptera: Aphididae) have been shown repeatedly (Honek 1991; Ponder et al. 2000; Awmack and Leather 2002; Hambäck et al. 2007), and plant nutritional quality has also been demonstrated to affect the performance of higher trophic levels (e.g. Omacini et al. 2001; Harvey et al. 2003; Bukovinszky et al. 2008; Garratt et al. 2010). Therefore, we hypothesized that the varying nitrogen supply of organic and conventional fields would influence trophic interactions and thereby biological pest control. In particular, we expected that: (1) the relatively higher nitrogen input in conventional fields would lead to increasing abundances of cereal aphids; (2) primary parasitoids (and subsequently secondary parasitoids) would respond to changes in resource quantity and/or host nutritional quality.

## Materials and methods

### Study site and insect sampling

The study was carried out in Northern Germany, in an area located 60 km north-east of Hamburg. The region is dominated by arable fields with intensive land use (approx. 60 %) embedded in a small-scale mosaic of woodlands (approx. 21 %), grasslands (14 %), hedges, hedgerows and other semi-natural habitats (approx. 2 %) (DLR 2009). We analysed a total of ten organically managed and eight conventional winter wheat fields using data collected for the period 2004–2007, representing an unbalanced design (organically managed fields: 2004, *n* = 4; 2005, *n* = 1; 2006, *n* = 2; 2007, *n* = 3; conventional fields: 2004, *n* = 2; 2005, *n* = 2; 2006, *n* = 2; 2007, *n* = 2). All samples were taken from different fields in different years. Conventional wheat fields were characterized by applications of 180–200 kg nitrogen/ha, while organically managed wheat fields were cultivated after legumes had been harvested as the preceding crop. Our plant analyses revealed that conventionally grown winter wheat had higher nitrogen content (%) than organically grown winter wheat (tillers at wheat flowering: 1.9 ± 0.2 vs. 1.1 ± 0.1 %, *n* = 1 sample per field; grains at wheat milk-ripening: 2.8 ± 0.6 vs. 2.1 ± 0.4 %, *n* = 1 sample per field; straw at wheat milk-ripening: 1.5 ± 0.2 vs. 0.8 ± 0.3 %, *n* = 1 sample per field, grains at harvest: 2.3 ± 0.1 vs. 1.9 ± 0.2 %, *n* = 1 sample per field; straw at harvest: 1.0 ± 0.3 vs. 0.4 ± 0.1, *n* = 1 sample). Each sample consisted of 60 tillers (subdivided in 12 sub-samples of five tillers). Total nitrogen (% dry mass) was analysed according to Dumas (1981) using a CNS-2000 elemental analyzer (LECO, St. Joseph, MI). The BBCH-code (Lancashire et al. 1991) was used for characterizing the growth stages of wheat plants. In 2004 and 2005, conventional winter wheat fields were sprayed with aphid-specific insecticides (“Karate Zeon”, 75 ml/ha) at BBCH 73 (beginning of milk-ripening stage). The maximum distance between fields was 2.6 km.

During BBCH 51 (beginning of inflorescence emergence) and BBCH 89 (end of ripening), aphids and parasitized aphids (mummies) were quantified visually at 4-day sampling intervals, with a total of 9–12 sampling dates per field and year. Each field sample consisted of 24 sub-samples of five tillers from different locations along a transect (=120 tillers per sampling date). Fields sprayed with aphid-specific insecticides were sampled 4 days after application. Mummies were transferred to the laboratory for rearing and identification of primary and secondary parasitoids. All aphids and parasitoids were identified to the species level using descriptions provided in the literature [Electronic Supplementary Material (ESM) 1]. We also recorded the abundance of vegetation-dwelling predators (Coccinellidae, Syrphidae, Chrysopidae), but predator densities in both organically managed and conventional fields were generally low (<0.1 individuals per 120 tillers) and therefore may not be expected to have significantly influenced aphid–parasitoid interactions.

### Calculation of quantitative food web metrics

In our investigation of a four-trophic level system, we resolved food webs to the genus level of insects because secondary parasitoids cannot be linked to primary parasitoid species but to the genus level of primary parasitoids by using typical cocoon characteristics (shape, colour) (Powell 1982). We calculated a quantitative measure of interaction evenness (IE) following Tylianakis et al. (2007). Weighted, quantitative versions of generality (G), vulnerability (V) and link density (LD) based on Shannon’s (1948) information theory were calculated following Bersier et al. (2002). These calculations are not as straightforward as the traditionally applied qualitative descriptors. Quantitative food web metrics, however, have been found to be much more robust to variations in sampling effort and are, therefore, more reliable for between-food web comparisons (Banasek-Richter et al. 2004). Quantitative interaction evenness is the evenness in the distribution of trophic links—i.e. it refers to the evenness of interaction frequencies. Quantitative generality is the weighted ratio of host taxa per consumer (i.e. the quantitative counterpart of the qualitative descriptor: mean number of host taxa per consumer), while quantitative vulnerability is the weighted ratio of consumer taxa per host (i.e. the quantitative counterpart of the qualitative descriptor: mean number of consumer taxa per host). Quantitative link density is a measure of connectivity and refers to both consumers and hosts; it is calculated as the average of quantitative vulnerability and generality (for details on calculations of quantitative food web metrics, see ESM 2). Both quantitative interaction evenness and link density are attributes of food web structure that are thought to confer stability or increased function to a system (Tylianakis et al. 2010). Food web metrics were calculated separately for three plant growth stages (flowering period: BBCH 51–69; milk-ripening period: BBCH 70–79; peak ripening period: BBCH 80–89) by pooling the abundances of aphids and parasitized aphids (mummies) across three to four sampling dates per field, respectively.

### Statistical analysis

The effects of farming practices on food web metrics were tested using general linear models (GLMs), with farming system as a fixed factor and host and consumer richness as covariates; food web metrics were log-transformed to meet the assumptions of the models. To account for the variability in aphid and parasitoid abundance between years and fields, we calculated the predicted values of each food web metric to assign weights to each analysis. We thereby used the inverse of the square of these predicted values following Neter et al. (1996). The data were analysed separately with regard to three plant growth stages (flowering period: BBCH 51–69; milk-ripening period: BBCH 70–79; peak ripening period: BBCH 80–89) for aphid–primary parasitoid food webs and primary parasitoid–secondary parasitoid food webs, respectively. This approach of analysing temporal sequences appears to be most suitable for aphid–parasitoid interactions, as aphid population development is known to show distinct dynamics over time (compare Thies et al. 2011). The variation in host and consumer richness [log (*X* + 1)-transformed] between organically managed and conventional fields was tested by a one-way analysis of variance (ANOVA). In aphid–primary parasitoid food webs, aphids represented the hosts and primary parasitoids the consumers. In primary parasitoid–secondary parasitoid food webs, primary parasitoids represented the hosts and secondary parasitoids the consumers.

Differences in aphid and parasitoid density [log (*X* + 1)-transformed] as well as parasitism and hyperparasitism rates [arcsine ($\sqrt X$)-transformed] between organically managed and conventional fields were calculated by a one-way analysis of variance (ANOVA). Parasitism of aphids was calculated as the fraction of aphids attacked (mummies) to total aphids, and hyperparasitism was calculated as the fraction of secondary parasitoids to mummies. Aphid mummies do not reflect exact parasitism rates, which are generally expected to be higher (Kuo-Sell and Eggers 1987). Mummy formation (i.e. the time from parasitization to mummification) takes about 1–2 weeks under field conditions (Höller et al. 1993), and our analyses therefore encompass this time lag in both organic and conventional fields. In addition, relationships between consumer and host abundance, food web metrics and parasitism/hyperparasitism rates, as well as the relationships between parasitism and hyperparasitism, respectively, and relative host abundance were analysed using linear regressions. All analyses were conducted using Statistica 8.0 (StatSoft, Tulsa, OK).

## Results

### Community composition

The densities of cereal aphids and of their primary and secondary parasitoids are summarized in Table 1. Aphid communities in organically managed fields were dominated by *S. avenae* (>96 % of all aphids), while *M. dirhodum* and *R. padi* were comparatively more abundant in conventional fields (together >79 % of all aphids); there was only little differences in relative abundance across the season for all aphids. Total aphid densities did not differ between organically managed and conventional fields (*P* values at all BBCH stages >0.05), and there were also no significant differences in the density of *S. avenae* (*P* values at all BBCH stages >0.05) due to the high variability. The densities of both *M.* *dirhodum* and *R. padi*, however, were significantly higher in conventional fields than in organically managed ones at flowering (*M.* *dirhodum*: *F*_1,16_ = 18.8, *P* < 0.001; *R. padi*: *F*_1,16_ = 44.7, *P* < 0.001), at milk-ripening (*M.* *dirhodum*: *F*_1,16_ = 17.9, *P* < 0.001; *R. padi*: *F*_1,16_ = 67.4, *P* < 0.001), and at peak ripening (*M.* *dirhodum*: *F*_1,16_ = 24.2, *P* < 0.001; *R. padi*: *F*_1,16_ = 92.3, *P* < 0.001).

**Table 1:** Density of cereal aphids and their primary and secondary parasitoids (individuals per 120 tillers) in wheat fields at different plant growth stages

Taxa	Organically managed fields			Conventional fields		
	Flowering stage	Milk-ripening stage	Peak ripening stage	Flowering stage	Milk-ripening stage	Peak ripening stage
Aphids						
Total aphids	284.6 ± 104.1	1,822.6 ± 561.4	315.5 ± 135.4	334.6 ± 71.7	2253.7 ± 596.9	532.9 ± 163.5
* Sitobion avenae*	273.4 ± 104.1	1,779.7 ± 562.7	308.5 ± 135.2	69.9 ± 34.8	444.0 ± 102.1	91.0 ± 18.4
* Metopolophium dirhodum*	7.2 ± 2.9	36.8 ± 12.3	5.0 ± 2.7	133.4 ± 43.9	1030.4 ± 357.8	274.8 ± 143.2
* Rhopalosiphum padi*	3.9 ± 1.3	6.1 ± 1.4	2.1 ± 0.8	131.7 ± 35.7	779.3 ± 275.9	167.2 ± 45.2
Primary parasitoids						
Total mummies	0.4 ± 0.2	5.9 ± 1.1	6.0 ± 1.9	1.0 ± 0.4	4.4 ± 1.1	3.6 ± 1.4
* Aphidius* spp.	0.2 ± 0.1	4.5 ± 1.3	5.2 ± 1.9	0.8 ± 0.4	3.3 ± 1.0	2.3 ± 1.3
* Trioxys* spp.	0	0	0	0	<0.1	0
* Ephedrus* spp.	0.2 ± 0.2	0.9 ± 0.3	0.5 ± 0.3	0.1 ± 0.1	0.3 ± 0.1	0.9 ± 0.7
* Toxares* spp.	0	0	0	0	0	0
* Praon* spp.	<0.1	0.5 ± 0.2	0.2 ± 0.2	0.1 ± 0.1	0.8 ± 0.3	0.1 ± 0.1
* Aphelinus* spp.	0	0	0.1 ± 0.1	0	<0.1	0.3 ± 0.1
Secondary parasitoids						
Total mummies	0.2 ± 0.1	2.9 ± 1.1	12.4 ± 6.1	0.2 ± 0.1	3.5 ± 1.1	7.4 ± 2.8
* Alloxysta* spp.	<0.1	0.1 ± 0.1	1.4 ± 1.1	<0.1	0.5 ± 0.2	0.1 ± 0.1
* Phaenoglyphis* spp.	<0.1	0.2 ± 0.1	1.9 ± 1.4	0	0.3 ± 0.2	0.3 ± 0.2
* Dendrocerus* spp.	0.1 ± 0.1	1.5 ± 0.6	4.7 ± 2.8	<0.1	0.8 ± 0.4	1.8 ± 0.6
* Aphanogmus* spp.	0	0	0	0	<0.1	0
* Asaphes* spp.	<0.1	0.9 ± 0.4	3.3 ± 0.7	0.1 ± 0.1	1.7 ± 0.5	4.7 ± 2.1
* Pachyneuron* spp.	0	0.2 ± 0.2	1.1 ± 0.7	0	0.1 ± 0.1	0.3 ± 0.3
* Coruna* spp.	0	0	<0.1	0	0.1 ± 0.1	0.2 ± 0.1

Data are presented as arithmetic means ± standard error (SE) for both organically managed fields (*n* = 10) and conventional fields (*n* = 8)

**Fig. 1:**
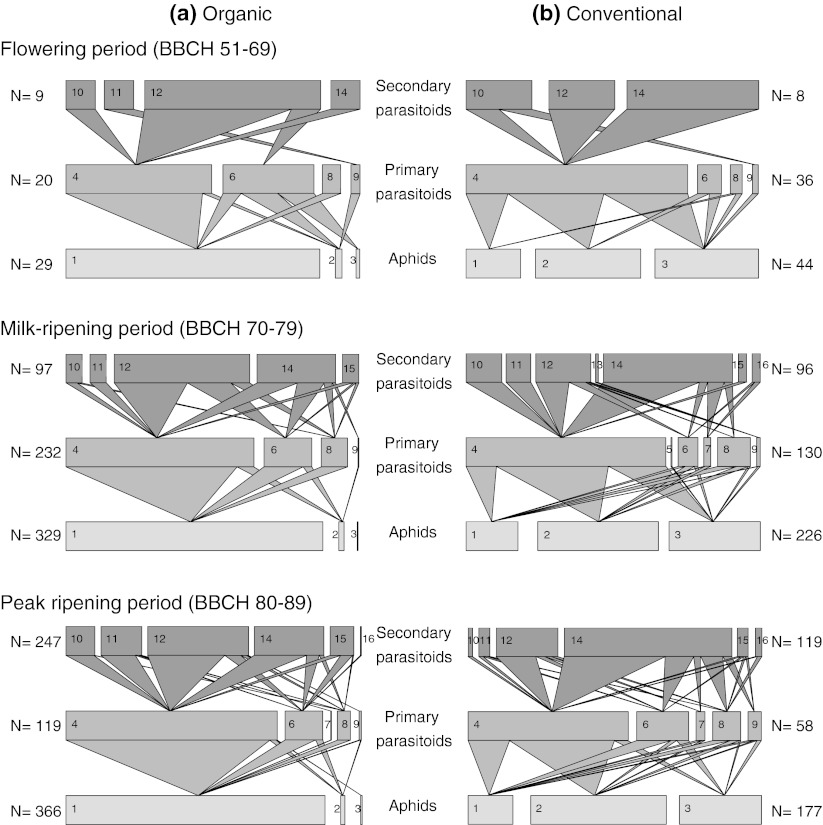
Quantitative aphid–parasitoid webs for organically managed fields (**a**) and conventional fields (**b**) at different plant growth stages. Aphid and primary and secondary parasitoid order is consistent across webs, showing the relative proportions of aphid, parasitoid and hyperparasitoid genera. Relative aphid abundances are represented by the *lower bars*, primary parasitoid abundances by the *middle bars*, and secondary parasitoid abundances by the *upper bars*. *Link widths* indicate relative frequencies of each trophic interaction. The data are pooled across all fields and replicates in terms of each plant growth stage. The number of individuals (*N*) is given for aphids, primary parasitoids and secondary parasitoids. Taxa codes: *1* *Sitobion avenae*, *2* *Metopolophium dirhodum*, *3* *Rhopalosiphum padi*, *4* *Aphidius* sp., *5* *Trioxys* sp., *6* *Ephedrus* sp., *7* *Toxare*s sp., *8* *Praon* sp., *9* *Aphelinus* sp., *10* A*lloxysta* sp., *11* *Phaenoglyphis* sp., *12* *Dendrocerus* sp., *13* *Aphanogmus* sp., *14* *Asaphes* sp., *15* *Pachyneuron* sp., *16* *Coruna* sp.

Primary parasitoid communities in both farming systems were dominated by a single genus, *Aphidius* (Fig. 1). The relative abundance of *Aphidius* increased from 55 % at flowering to 80 % at peak ripening in organically managed fields, but decreased from 84 % at the flowering period to 61 % at peak ripening in conventional fields. Secondary parasitoid communities were mainly represented by the mummy parasitoid genera *Dendrocerus*, *Asaphes*, *Pachyneuron* and *Coruna*. In conventional fields, their relative abundances increased from 75 % at flowering to 94 % at peak ripening, while the relative abundances of the true hyperparasitoid genera, *Alloxysta* and *Phaenoglyphis*, decreased from 25 % at flowering to 6 % at peak ripening. In organically managed fields, the proportion of mummy and true hyperparasitoid genera did not consistently increase or decrease over time (Fig. 1a). Total primary and secondary parasitoid abundances did not differ between organically managed and conventional fields (*P* values at all BBCH stages >0.05). There were also no differences between organically managed and conventional fields in the abundance of single primary and secondary parasitoid genera, respectively (*P* values at all BBCH stages >0.05), except for those of the secondary parasitoid *Coruna* that occurred sporadically in some conventional fields at the milk-ripening period (*F*_1,16_ = 5.2, *P* = 0.037). Overall, in organically managed fields, aphids were attacked by five genera of primary parasitoids and by six genera of secondary parasitoids. In conventional fields, aphids were attacked by six genera of primary parasitoids and by seven genera of secondary parasitoids. The higher incidence of hymenopterous taxa in conventional fields was due to the sporadic occurrence of the primary parasitoid *Trioxys* and the secondary parasitoid *Aphanogmus*.

Aphid abundance and primary parasitoid abundance were positively correlated at milk-ripening (*R* = 0.62, *P* = 0.006) and at peak ripening (*R* = 0.59, *P* = 0.011). Primary parasitoid abundance and secondary parasitoid abundance were also positively correlated at peak ripening (*R* = 0.48, *P* = 0.047).

### Aphid–primary parasitoid and primary parasitoid–secondary parasitoid food web metrics

**Fig. 2:**
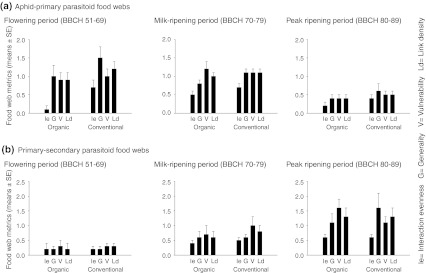
Quantitative food web metrics of aphid–primary parasitoid webs (**a**) and primary parasitoid–secondary parasitoid webs (**b**) at different plant growth stages.* Bars* represent arithmetic means ± standard error (SE) for both organically managed fields (*n* = 10) and conventional fields (*n* = 8). *Ie* interaction evenness, *G* generality, *V* vulnerability, *Ld* link density

Our inspection of quantitative food webs (Fig. 1) and their metrics (Fig. 2) revealed striking differences in the structure of trophic interactions between organically managed and conventional fields. Quantitative interaction evenness and quantitative vulnerability in aphid–primary parasitoid food webs were significantly higher in conventional than organically managed fields at flowering and/or milk-ripening and peak ripening (Table 2). Interaction evenness was significantly increased by both host and consumer richness, and vulnerability was increased by host richness (Table 2). In the aphid–primary parasitoid food webs, host genera richness was significantly higher in conventional than organically managed fields at milk-ripening (*F*_1,16_ = 41.5, *P* < 0.001), while consumer genera richness did not vary between farming systems (*P* values at all BBCH stages >0.05) (Table 3).

**Table 2:** Effects of farming system on quantitative food web metrics at different plant growth stages

Quantitative food web metrics	Flowering	Milk-ripening	Peak ripening
Aphid–primary parasitoid food webs			
Interaction evenness			
Farming system	26.4***	38.9***	ns
Host richness	41.4***	8.5*	9.1**
Consumer richness	ns	46.1***	6.1*
Generality			
Farming system	ns	ns	ns
Host richness	10.9*	ns	19.7***
Consumer richness	ns	ns	ns
Vulnerability			
Farming system	ns	ns	6.5*
Host richness	ns	12.3**	28.4***
Consumer richness	ns	6.4*	ns
Link density			
Farming system	ns	ns	ns
Host richness	8.1*	ns	25.0***
Consumer richness	ns	ns	ns
Primary parasitoid–secondary parasitoid food webs			
Interaction evenness			
Farming system	ns	6.2*	ns
Host richness	ns	21.8***	9.1**
Consumer richness	10.2*	11.6**	5.3*
Generality			
Farming system	ns	17.4***	5.2*
Host richness	19.6**	86.7***	128.2***
Consumer richness	ns	ns	ns
Vulnerability			
Farming system	ns	11.2**	12.2**^a^
Host richness	23.2***	54.7***	33.0***
Consumer richness	ns	19.2***	25.4***
Link density			
Farming system	ns	15.9**	ns
Host richness	21.7**	92.7***	79.1***
Consumer richness	ns	19.1***	8.8*

Most results are highly significant. The application of the Bonferroni method therefore does not change the main results. *F* values and levels of significance derived from general linear models (GLMs)

ns, Not significant

^ a^ *F* values were higher in the organic farming system; for all other metrics, *F* values were higher in the conventional farming system. Organically managed fields (*n* = 10), conventional fields (*n* = 8)

**Table 3:** Host and consumer richness at different plant growth stages

	Organically managed fields			Conventional fields		
	Flowering stage	Milk-ripening stage	Peak ripening stage	Flowering stage	Milk-ripening stage	Peak ripening stage
Aphid–primary parasitoid food webs						
Host taxa	1.3 ± 0.3	1.3 ± 0.2	1.0 ± 0.2	2.2 ± 0.3	2.8 ± 0.2***	1.6 ± 0.3
Consumer taxa	1.1 ± 0.2	2.3 ± 0.3	1.4 ± 0.3	1.7 ± 0.4	2.8 ± 0.2	1.5 ± 0.4
Primary parasitoid–secondary parasitoid food webs						
Host taxa	0.6 ± 0.3	1.9 ± 0.4	2.1 ± 0.3	0.8 ± 0.3	2.3 ± 0.4	2.5 ± 0.5
Consumer taxa	0.7 ± 0.4	2.9 ± 0.4	3.4 ± 0.4	1.2 ± 0.5	3.6 ± 0.6	2.6 ± 0.7

Data are presented as the arithmetic means ± SE for organically managed fields (*n* = 10) and conventional fields (*n* = 8). Levels of significance derived from a one-way analysis of variance (ANOVA). In aphid–primary parasitoid food webs, aphids represent the hosts and primary parasitoids the consumers. In primary parasitoid–secondary parasitoid food webs, primary parasitoids represent the hosts and secondary parasitoids the consumers

*** *P* < 0.001

In contrast to aphid–primary parasitoid food webs, quantitative vulnerability in primary parasitoid parasitoid–secondary parasitoid food webs was significantly higher in organically managed than conventional fields at peak ripening (Table 2). Higher values of vulnerability in the organically managed fields, however, developed only later in the season (at peak ripening), while this metric was still higher in conventional fields at milk-ripening. In accordance with aphid–primary parasitoid food webs, quantitative interaction evenness was significantly higher in conventional than organically managed fields at milk-ripening. In addition, quantitative generality was significantly higher in conventional than organically managed fields at milk-ripening and peak ripening, and quantitative link density was significantly higher in conventional than organically managed fields at milk-ripening. Interaction evenness, vulnerability and link density were significantly increased by both host and consumer richness, and generality was increased by host richness (Table 2). In primary parasitoid–secondary parasitoid food webs, the richness of host and consumer genera did not differ between farming systems (*P* values at all BBCH stages >0.05) (Table 3).

### Parasitism and hyperparasitism

The parasitism rate was generally low during the population development of aphids and did not differ between farming systems at flowering (organic 0.8 ± 0.4, conventional 0.6 ± 0.2) and at milk-ripening (organic 2.3 ± 0.8, conventional 1.1 ± 0.4) (*P* values at both BBCH stages >0.05), but was significantly higher in organically managed than conventional fields at peak ripening when aphid populations were broken down (organic 28.8 ± 7.1, conventional 6.6 ± 1.7; *F*_1,16_ = 9.1, *P* = 0.008). The hyperparasitism rate did not differ between farming systems at flowering (organic 16.1 ± 8.9, conventional 20.5 ± 10.5), at milk-ripening (organic 33.6 ± 8.5, conventional 45.8 ± 7.6) and at peak ripening (organic 68.5 ± 7.5, conventional 62.7 ± 9.1; *P* values at all BBCH stages >0.05).

In aphid–primary parasitoid food webs, parasitism rate did not correlate with food web metrics. By contrast, in primary parasitoid–secondary parasitoid food webs, the hyperparasitism rate correlated positively with quantitative interaction evenness at milk-ripening (*R* = 0.82, *P* < 0.001), with quantitative generality at milk-ripening (*R* = 0.91, *P* < 0.001) and peak ripening (*R* = 0.79, *P* < 0.001), with quantitative vulnerability at milk-ripening (*R* = 0.82, *P* < 0.001) and peak ripening (*R* = 0.54, *P* = 0.020) and with quantitative link density at milk-ripening (*R* = 0.89, *P* < 0.001). The parasitism and hyperparasitism rates by the most abundant parasitoid and hyperparasitoid genera, respectively, were neither correlated with relative abundances of their host genera within farming systems (organically managed fields: *Aphidius*–*S. avenae*, *R* = 0.50; *Dendrocerus*–*Aphidius*, *R* = 0.35; *Asaphes*–*Aphidius*, *R* = 0.27; all *P* values >0.05; conventional fields: *Aphidius*–*M. dirhodum*, *R* = 0.30; *Aphidius*–*R. padi*, *R* = 0.62; *Dendrocerus*–*Aphidius*, *R* = 0.05; *Asaphes*–*Aphidius*, *R* = 0.04; all *P* values >0.05), nor between farming systems (all *P* values > 0.05), showing density-independent parasitism and hyperparasitism.

## Discussion

### Effects of community composition on food web complexity

Our analyses of quantitative food webs revealed marked changes in the interaction structure of cereal aphids and their primary and secondary parasitoids that were related to farming practices. Variation in relative aphid and parasitoid abundance between organically managed and conventional fields also contributed to the explanation of food web structure. Aphid communities in organically managed fields almost exclusively consisted of a single ear-colonizing species (*S. avenae*), while conventional fields were mainly infested by leaf-colonizing aphids (*M. dirhodum*, *R. padi*). Nitrogen contents were lower in organically grown than conventional wheat plants, and *S. avenae* has been documented to be not affected or even positively affected by a low nutritional plant quality, while *M. dirhodum* and *R. padi* are known to be positively influenced by high nitrogen applications (e.g. Hasken and Poehling 1995; Duffield et al. 1997; Hambäck et al. 2007). Applications of insecticides, such as in our study, may have reduced species abundances, but they did result in an even higher food web complexity in conventional fields. Therefore, nitrogen differences appear to be a driving force behind food web interaction structures.

At a higher trophic level, food web interactions in the organically managed fields were dominated by a single trophic interaction of the major primary parasitoid, *Aphidius*, parasitizing the dominating aphid *S. avenae*, whereas the availability of the aphids *M. dirhodum* and *R. padi* in conventional fields resulted in more evenly distributed links of *Aphidius* parasitism. Hence, the high interaction evenness in conventional fields indicates bottom–up-induced food web changes that propagate to the next trophic level, with higher values of quantitative generality and link density. Such effects on higher trophic levels mediated through bottom–up trophic cascades have proven to be widespread in parasitoid–host communities (Hawkins 1992). Moreover, a high evenness of interaction frequencies provided by several trophic links may contribute to enhance the stability of antagonistic networks, while only a few strong trophic interactions, such as those in our organically managed fields, may be expected to have destabilizing effects (McCann et al. 1998; McCann 2000).

The functioning of trophic cascades owing to qualitative changes in the plant resource at the bottom of the food webs was reflected by higher values of quantitative generality (the weighted ratio of host taxa per consumer) in the conventional fields. In this process, primary parasitoid–secondary parasitoid interactions were characterized by a wider host taxa spectrum of generalistic mummy parasitoids (see Godfray 1994), whose abundances continuously increased from the flowering stage to the peak ripening stage. Consequently, higher values of generality in the conventional fields were caused by a higher number of inflows—i.e. they were due to a high number of interactions between primary parasitoid taxa and secondary parasitoids (for the calculation of quantitative generality, see ESM 2). In the aphid–primary parasitoid food webs, the higher values of quantitative vulnerability (the weighted ratio of consumer taxa per host) in the conventional fields were caused by a higher number of outflows—i.e. they were due to a high number of interactions between primary parasitoid taxa and aphids, relying on an increased availability of alternative aphid taxa. Primary parasitoid–secondary parasitoid interactions, however, were characterized by high relative abundances of *Aphidius* in both conventional and organically managed fields that led to an increase in the magnitude of outflows—i.e. they resulted in more evenly distributed interactions between secondary parasitoid taxa and primary parasitoids (for the calculation of quantitative vulnerability, see ESM 2). Differences in quantitative link density (the weighted average of vulnerability and generality) can be expected in situations when a high generality (diversity of inflows) is not counterbalanced by a low vulnerability (diversity of outflows). Such opposing trends of vulnerability and generality were apparent in both farming systems, in that a higher vulnerability was associated with a lower generality in the organically managed fields, and a lower vulnerability was associated with a higher generality in the conventional fields (see Fig. 2). This is likely to explain the lack of stronger effects in terms of quantitative link density.

### Parasitism and hyperparasitism rates

Parasitism rates were below values which have been suggested to be effective for biological aphid control by parasitoids (Hawkins and Cornell 1994; Giller et al. 1995) during aphid population development at the flowering and milk-ripening stages. Despite the lower food web complexity, parasitism rates were higher in the organically managed than conventional fields later in the season (at peak ripening). The higher rates of aphid parasitism in the organically managed fields might be related to a preference of parasitoids for *S. avenae*: the colonization of wheat fields by *S. avenae* is temporally more closely related to the colonization of the main parasitoid, *Aphidius*, as this aphid reaches its population peak regularly later than *M. dirhodum* and *R. padi* (Ankersmit and Carter 1981; Gianoli 2000). Furthermore, *S. avenae* predominantly feeds on ears and upper leafs and therefore might be more easily accessible to most primary parasitoid taxa than the leaf-colonizing aphid species due to its exposed feeding site. Attack rates by single parasitoid and hyperparasitoid genera, however, did not correlate with the relative abundances of their hosts, thereby indicating that parasitism was not influenced by relative host abundances or host accessibility. Our findings are in line with those of Hawkins et al. (1999) and Rodriguez and Hawkins (2000) who pointed out that top–down control in most host-parasitoid systems is likely in situations when one or a few key species in simplified food webs dominate the trophic interactions, and is not a result of the diversity per se. The role of species identity for the functioning of biological control processes is not well understood, but species may be important in cereal aphid control as higher parasitism rates were found to be related to low quantitative interaction evenness resulting from host specialization of the major aphid parasitoid, *Aphidius*. Therefore, our results suggest that food web complexity and ecosystem functioning in aphid–parasitoid webs are negatively linked (compare Montoya et al. 2003; Gagic et al. 2011). Moreover, diverse parasitoid communities have been shown to enhance aphid suppression by complementary resource use (Finke and Snyder 2008) and may consequently be of minor importance in our organically managed fields that were less aphid-rich. The higher floral diversity in organically managed fields may have supported aphid parasitism later in the season due to a higher availability of alternative food resources (Langer 2001; Vollhardt et al. 2010; but see Roschewitz et al. 2005), while parasitoid species richness does not appear to have benefitted (compare Vollhardt et al. 2008). Apparently, aphid parasitoids are able to cope with their resources in simple environments, such as conventionally managed fields. The application of insecticides in conventional fields (in 2 out of 4 years) may have decreased aphid and parasitoid abundances at peak ripening, while the recovery and recolonization of fields can occur within a few days (Langhof et al. 2003).

Hyperparasitism rates reached high values of >60 % late in the season (at peak ripening), when aphid population densities break down owing to decreasing resource quality. The role of secondary parasitoids is not well understood, but they have been shown to be strongly linked to their host and therefore may disrupt the potential control of aphids by primary parasitoids in the following year (Sunderland et al. 1997; Rosenheim 1998). In our study, hyperparasitism was positively related to food web complexity, with correlations being strongest at the milk-ripening stage, which is the time of maximum aphid abundance. Therefore, weak interactions between aphids and primary parasitoids (i.e. low parasitism rates) may induce effects that cascade to the next trophic level and ultimately foster complex interaction structures between primary and secondary parasitoids. Interestingly, in structurally complex landscapes, a low food web complexity (quantitative generality, link density and interaction diversity) in conjunction with high aphid parasitism rates have been reported to have no consequences for higher trophic level interactions between primary and secondary parasitoids (Gagic et al. 2011). This result suggests that the structure of feeding interactions may change in situations when primary parasitoids play a stronger role, such as in structurally complex landscapes. However, an increased taxonomic resolution based on the analysis of aphid–parasitoid species interactions via molecular approaches (Traugott et al. 2008; Macfadyen et al. 2009b) is likely to contribute to a better understanding of multi-trophic interactions and may also reveal hidden interactions, such as facultative hyperparasitism by primary parasitoids or trophic loops within the guild of mummy parasitoids.

In conclusion, the structure of feeding interactions between cereal aphids and their parasitoids and hyperparasitoids in organically managed and conventional fields has to be reconsidered. In our study, trophic interactions between cereal aphids and primary parasitoids appeared to be controlled by resource-based forces (bottom–up) that can trigger strong effects on interactions at the next trophic level, thereby enhancing food web complexity in conventionally managed winter wheat fields. On the other hand, top–down control tended to be higher in simplified food webs, thus supporting the notion that species identity plays a role in biological pest control. The marked changes in food web structure between the farming systems in our study suggest that the functioning of a variety of taxa can largely differ and be misinterpreted in studies that do not quantify trophic interactions. More field studies should compare food web interactions in organically managed and conventional fields located in landscapes differing in structural complexity in order to differentiate the effects of agricultural intensification at local and landscape scales, as well as the biotic and abiotic factors that determine population densities.

## Electronic supplementary material

Below is the link to the electronic supplementary material.

Supplementary material 1 (DOC 41 kb)
