# Supplementary material for: Farming practices change food web structures in cereal aphid–parasitoid–hyperparasitoid communities
Source: Oecologia. 2012 Jun 27;171(1):249–59. doi: 10.1007/s00442-012-2387-8 (PMC3538122; doi:10.1007/s00442-012-2387-8)
Supplement: Supplementary file 1 — Supplementary material 1 (DOC 41 kb) [file 442_2012_2387_MOESM1_ESM.doc]

**Electronic Supplementary Material**

**Farming practices change food web structures in cereal aphid-parasitoid-hyperparasitoid communities**

Katharina Lohaus, Stefan Vidal & Carsten Thies

Author for correspondence: kalohaus@agr.uni-goettingen.de

**ESM 1**

**Literature for identification of aphid and parasitoid species**

Aphids

Heie, O.E. (1986): The Aphidoidea (Hemiptera) of Fennoscandia and Denmark. III. Family Aphididae: subfamily Pterocommatinae & tribe Ahidini of subfamily Aphidinae. Fauna Entomologica Scandinavica 17. E.J. Brill/Scandinavian Science Press Ltd., Leiden.

Olsen, C.E., Pike, K.S., Boydston, L. & Allison, D. (1993): Keys for identification of apterous viviparae and immatures of six small grain aphids (Homoptera.Aphididae). Journal of Economic Entomology 86, 137-148.

Primary parasitoids (Braconidae)

Mackauer, M. (1959): Die europäischen Arten der Gattungen *Praon* und *Areopraon* (Hymenoptera: Braconidae, Aphidiinae). Beiträge zur Entomologie 9, 810-865.

Pike, K.S., Stary, P., Miller, T., Allison, D., Boydston, L., Graf, G. & Gillespie, R. (1997): Small-grain aphid parasitoids (Hymenoptera: Aphelinidae and Aphidiidae) of Washington: Distribution, relative abundance, seasonal occurence, and key to known North American Species. Environmental Entomology 26, 1299-1311.

Tobias, V.I & Kiriyak, I.G. (1995): Familiy Aphididae. Keys to the insects of the European part of the USSR. Vol. III, Hymenoptera, part V (ed G.S. Medvedev), pp 387-507. Amerind Publishing Co. Pvt. Ltd., New Delhi.

Primary parasitoids (Aphelinidae)

Graham, M.W.R. de V. (1976): The British species of *Aphelinus* with notes and descriptions of other European Aphelinidae (Hymenoptera). Systematic Entomology 1, 123-146.

Secondary parasitoids (Alloxystidae)

Evenhuis, H.H. (1982): A study of Hartig`s Xystus species with type designations and new synonyms (Hymenopera: Cynipidae Alloxystinae and Charipinae). Spixiana 5, 19-29.

Evenhuis, H.H. & Barbotin, F. (1977): Studies on Cynipidae Alloxystinae 6. *Phaenoglyphis villosa* (Hartig) and *Alloxysta* *arcuata* (Kieffer). Entomologische Berichten. 37, 184-190.

Höller, C. (1988): Effizienzanalyse der Parasitoiden an Getreideblattläusen. PhD thesis, University of Kiel.

Secondary parasitoids (Megaspilidae and Ceraphronidae)

Alekseev, V.N. (1987): Superfamily Ceraphronoidea (Ceraphronoids). Keys to the insects of the European part of the USSR. Vol. III, Hymenoptera, part II (ed G.S. Medvedev), pp. 1213- 1257. Oxonian Press Pvt. Ltd., New Delhi.

Fergusson, N.D.M. (1980): A revision of the British species of *Dendrocerus* Ratzeburg (Hymenoptera: Ceraphronoidea) with a review of their biology as aphid hyperparasites. Bulletin of the British Museum of Natural History (Entomology) 41, 255-314.

Secondary parasitoids (Pteromalidae)

Graham, M.W.R. de V. (1969): The Pteromalidae of north-western Europe. Bulletin of the British Museum of Natural History (Entomology), Supplement 16, 1-908.

Kamijo, K. & Takada, H. (1973): Studies on aphid hyperparasites of Japan, II Aphid hyperparasites of the Pteromalidae occuring in Japan (Hymanoptera). Insecta matsumurana new series 2, 39-76.

Secondary parasitoids (Encyrtidae)

Tryapitsyn, V. A. (1987): Family Encyrtidae (Encyrtids). Keys to the insects of the European part of the USSR. Vol. III, Hymenoptera, part II (ed G.S. Medvedev), pp. 427-594. Oxonian Press Pvt. Ltd., New Delhi.

**ESM 2**

**Calculation of quantitative food web metrics**

We calculated a quantitative measure of interaction evenness (IE) following Tylianakis, Tscharntke & Lewis (2007) to account for differences in the evenness in the distribution of link magnitude (interaction frequencies):

where *p_i_* is the proportion of the total number of consumer-prey interactions (*N*) represented by interaction *i*.

Quantitative versions of generality, vulnerability and link density were calculated following Bersier, Banasek-Richter & Cattin (2002). For each taxon *k*, the food web metrics incorporate the biomass coming from its prey (*H_N_*, the diversity of inflows) and of that going to its consumers (*H_P_*, the diversity of outflows).

Column sum *b*•*k* and row sum *bk*• represent the total amount of biomass of inflows and outflows of taxon *k*, respectively, requiring an identical biomass of prey and consumers. The food web metrics are based on the “reciprocals” of *H_N_*,*_k_* and *H_P_*,*_k._*

Generality (G) and vulnerability (V) are defined as the average number of prey taxa per consumer and the average number of consumer taxa per prey, respectively. Their quantitative, weighted equivalents include information on the interaction strength of each link and were calculated as:

Therefore *G_q_* is increasing with an increasing number of prey taxa attacked by a consumer taxon *k*, expressed by *n_N_*,*_k_* (number of inflows) and with an increasing ratio of prey individuals attacked by a consumer taxon *k* to the overall individuals of prey, expressed by *b*•*k* / *b*•• (magnitude of inflows). *V_q_* is increasing with an increasing number of consumer taxa attacking a prey taxon *k*, expressed by *n_P_*,*_k_* (number of outflows) and with an increasing ratio of consumer individuals attacking a prey taxon *k* to the overall individuals of consumers, expressed by *bk*•/ *b*•• (magnitude of outflows).

Qualitative link density (LD) is the ratio of the number of trophic links to the number of taxa. A quantitative version of link density was calculated as:
